# Implementation phase of a multicentre prehospital telemedicine system to support paramedics: feasibility and possible limitations

**DOI:** 10.1186/1757-7241-21-54

**Published:** 2013-07-11

**Authors:** Sebastian Bergrath, Michael Czaplik, Rolf Rossaint, Frederik Hirsch, Stefan Kurt Beckers, Bernd Valentin, Daniel Wielpütz, Marie-Thérèse Schneiders, Jörg Christian Brokmann

**Affiliations:** 1 Department of Anaesthesiology, University Hospital Aachen, Aachen, Germany; 2 Emergency Medical Service, Fire Department, Aachen, Germany; 3 Institute of Information Management in Mechanical Engineering, RWTH Aachen University, Aachen, Germany; 4 Emergency Department, University Hospital Aachen, Aachen, Germany

**Keywords:** Telemedicine, Teleconsultation, Telepresence, Emergency medical service, Analgesia

## Abstract

**Background:**

Legal regulations often limit the medical care that paramedics can provide. Telemedical solutions could overcome these limitations by remotely providing expert support. Therefore, a mobile telemedicine system to support paramedics was developed. During the implementation phase of this system in four German emergency medical services (EMS), the feasibility and possible limitations of this system were evaluated.

**Methods:**

After obtaining ethical approval and providing a structured training program for all medical professionals, the system was implemented on three paramedic-staffed ambulances on August 1^st^, 2012. Two more ambulances were included subsequently during this month. The paramedics could initiate a consultation with EMS physicians at a teleconsultation centre. Telemedical functionalities included audio communication, real-time vital data transmission, 12-lead electrocardiogram, picture transmission on demand, and video streaming from a camera embedded into the ceiling of each ambulance. After each consultation, telephone-based debriefings were conducted. Data were retrieved from the documentation protocols of the teleconsultation centre and the EMS.

**Results:**

During a one month period, teleconsultations were conducted during 35 (11.8%) of 296 emergency missions with a mean duration of 24.9 min (SD 12.5). Trauma, acute coronary syndromes, and circulatory emergencies represented 20 (57%) of the consultation cases. Diagnostic support was provided in 34 (97%) cases, and the administration of 50 individual medications, including opioids, was delegated by the teleconsultation centre to the paramedics in 21 (60%) missions (range: 1–7 per mission). No medical complications or negative interpersonal effects were reported. All applications functioned as expected except in one case in which the connection failed due to the lack of a viable mobile network.

**Conclusion:**

The feasibility of the telemedical approach was demonstrated. Teleconsultation enabled early initiation of treatments by paramedics operating under the real-time medical direction. Teleconsultation can be used to provide advanced care until the patient is under a physician’s care; moreover, it can be used to support the paramedics who work alone to provide treatment in non-life-threatening cases. Non-availability of mobile networks may be a relevant limitation. A larger prospective controlled trial is needed to evaluate the rate of complications and outcome effects.

## Background

Emergency medical services (EMS) can differ significantly among developed countries. In many countries, only paramedics provide prehospital emergency care, whereas EMS physicians are dispatched to the emergency site in other countries. In Germany, a dual system for emergency response has been established with both paramedics and EMS physicians responding. Resources are dispatched by EMS dispatchers according to locally prepared guidelines and the dispatcher’s personal assessment of the situation. German paramedics provide care without a physician present for 50% of all emergency missions; an EMS physician is present for the remaining 50% (including emergency secondary transfers) [1]. As is the case in many countries, national laws and local regulations restrict the medications German paramedics may administer and the invasive procedures they may perform. Whenever a paramedic performs an invasive procedure or administers a medication, an EMS physician must be summoned additionally. Paramedics are not allowed to administer opioids without a delegation by a physician. A general delegation to the paramedics is not permitted.

According to the recommendations of the German Medical Association, an EMS physician should be dispatched in all potentially life threatening situations. Often, paramedics are the first to arrive on the scene, and a substantial amount of time may pass before an EMS physician arrives; therefore, certain treatments are often delayed.

Telemedical solutions are increasingly being used in acute care specialties. Previous research has clearly demonstrated that the transmission of a prehospital 12-lead electrocardiogram (ECG) and consultation with the on-call cardiologist can improve treatment processes and outcomes [2-4]. In cases of acute stroke, pre-notification of the admitting stroke centre using telemedical applications and the provision of inter-hospital teleconsultation have been shown to improve treatment intervals and functional patient outcomes [5-7]. In an earlier pilot project, we found that telemedically supported paramedics were able to perform advanced treatments in simulated emergencies while providing a quality of care that was similar to that provided by on-scene EMS physicians [8].

With this information in mind, we incorporated a telemedical system into clinical practise to provide support to paramedics at an emergency site and en route to a hospital during any type of emergency. The aim of this study was to analyse the feasibility and possible limitations of prehospital teleconsultation during the implementation phase of this system.

## Methods

This prospective, observational study was conducted within the research project entitled “Telemedical Rescue Assistance System” (TemRas). After approval by the local ethics committee (University Hospital Aachen Germany, registration number EK 191/11), the system was implemented on August 1^st^, 2012.

### System details and system in use

**Figure 1:**
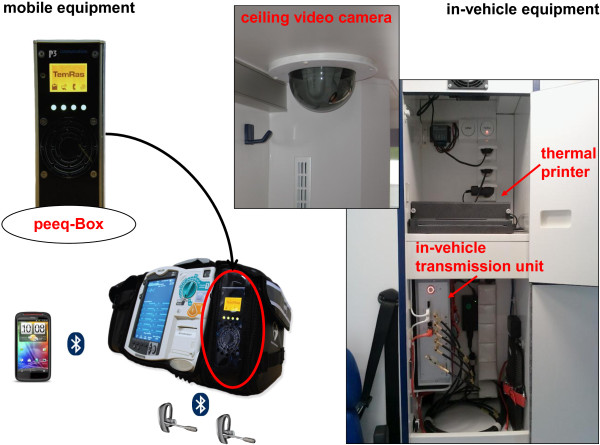
**Telemedical equipment on the ambulance.** Peeq-Box: mobile data and audio transmission unit.

**Figure 2:**
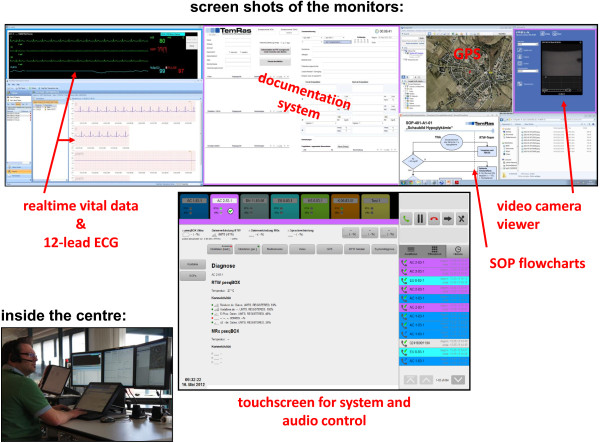
**Teleconsultation centre and screenshots of workstation.** SOP, standard operating procedure.

A pilot system for teleconsultation was developed as part of a previous project and tested in one physician-staffed ambulance [9-11]. Over a two-year period, a completely new technical system was designed and tested based on the experiences with the pilot system. Selected paramedic-staffed ambulances were telemedically equipped (Figure 1). A teleconsultation centre was established and staffed with experienced EMS physicians (i.e., tele-EMS physicians) (Figure 2). The paramedics used an MRx monitor-defibrillator unit (Philips Healthcare, Andover, MA, USA) connected to a portable data transmission unit (peeq-Box, P3 communications, Aachen, Germany). This uniquely designed transmission unit enabled simultaneous data transmission via three different mobile networks (2^nd^ and 3^rd^ generation). Audio communication was implemented using two headsets (Voyager Pro HD Plantronics, Santa Cruz, CA, USA) that were connected to the transmission unit. When the unit was located inside the ambulance, the data transmission switched to an in-vehicle transmission unit (up to five mobile networks; P3 communications, Aachen, Germany) that was connected to the roof antennas. Real-time vital data transmission (i.e., non-invasive blood pressure, numerical values and curves from a 3-lead rhythm-ECG, and pulse oximetry) was accomplished via remote activation in the teleconsultation centre. If required, 12-lead ECGs could also be transmitted by the paramedics. Moreover, still photographs taken with the built-in camera of a Smartphone (HTC Sensation XE, High Tech Computer Corporation, Taoyuan, Taiwan) and a video stream from a camera embedded in the ceiling of the ambulance (SNC-RZ 50P, Sony Electronics Inc., San Jose, CA, USA) could be transmitted. Secure data transmission was realized with encapsulated IP traffic over multiple radio links using virtual private networks. State of the art end-to-end encryption (AES 256) ensured further data security. In the teleconsultation centre, a proxy server unwrapped all data packages and restored the original application protocol.

On the telemedical workstation, the real-time vital data transmission was displayed using an IntelliVue Information Center (Philips Healthcare, Boeblingen, Germany), and 12-lead-ECGs were displayed on a HeartStart Telemedicine Viewer (Philips Healthcare, Andover, MA, USA). All other components in the teleconsultation centre (e.g., documentation software) were specifically developed as part of the research project. Standard operating procedures to enable guidelines-adherent treatment recommendations were developed for the following emergency situations: acute coronary syndrome (ACS), stroke, trauma and non-trauma analgesia, hypertension control, asthma, chronic obstructive pulmonary disease, and hypoglycaemia.

### Organisational setting

Five paramedic-staffed ambulances from four different EMS districts participated (Table 1). The regulations of the conveyor required that all ambulances were located in the state of Northrhine Westphalia (Germany). Four districts were chosen based on pre-existing cooperation with an emphasis on ensuring a mixture of urban and non-urban ambulances. The deployment of the ambulances and the EMS physician-staffed response units by the four local EMS dispatch centres continued as normal. The teleconsultation centre was operational during weekdays from 7:30 am to 4:30 pm. Two tele-EMS physicians could be contacted by the paramedic teams, as needed. No guidelines were given to define when teleconsultation was mandatory. The decision to use this additional service was at the discretion of the paramedics based on medical necessity and the availability of an on-scene EMS physician. If the patient was awake and oriented, verbal consent was obtained for the teleconsultation. After the initiation of an audio connection by the paramedics, the tele-EMS physician used software-based documentation software with integrated checklists to perform a structured query about the patient’s condition: The ABCDE- (airway, breathing, circulation, disability, exposure) and the SAMPLE-scheme (symptoms, allergies, medication, past medical history, last meal, events) should be filled out. Based on this assessment the tele-EMS physician then delegated administration of medications and/or performance of procedures to the paramedics. After each telemedically assisted mission was completed, telephone-based debriefings between the paramedics and the tele-EMS physician to discuss communication style, medical necessity, technical issues, and possible adverse events were conducted. A standardized report for any potential problems was completed daily by the tele-EMS physicians.

**Table 1:** Demographics and structure of the participating districts

	**Aachen (city)**	**Heinsberg (county)**	**Düren (county)**	**Euskirchen (county)**
population	248137	256546	267712	190591
area	160.8 km^2^	628.0 km^2^	941.4 km^2^	1248.7 km^2^
ambulances 24 hrs (telemedically equipped^a^)	6 (2)	7 (1)	11 (1)	9 (1)
ambulances daytime	2	3	2	2
EMS physician units	2+1^b^	4	4+1^c^	3
ambulance emergency missions / year	22984	14346	20302	15108
EMS physician missions / year	7898	7786	9057	5317
hospitals	4	4	5	3
- stroke units	1	1	1	1
- level 1 trauma centre	1	-	-	-
- 24 hrs cardiac cath lab	1	1	1	1

EMS, emergency medical service; hrs, hours.

^a^ telemedically equipped 24 hours, operation of teleconsultation centre 7.30 am – 4.30 pm.

^b^ additional EMS physician at home can be picked up by the fire department.

^c^ daytime EMS physician unit 7 am – 4 pm.

### Study flow and participants

**Figure 3:**
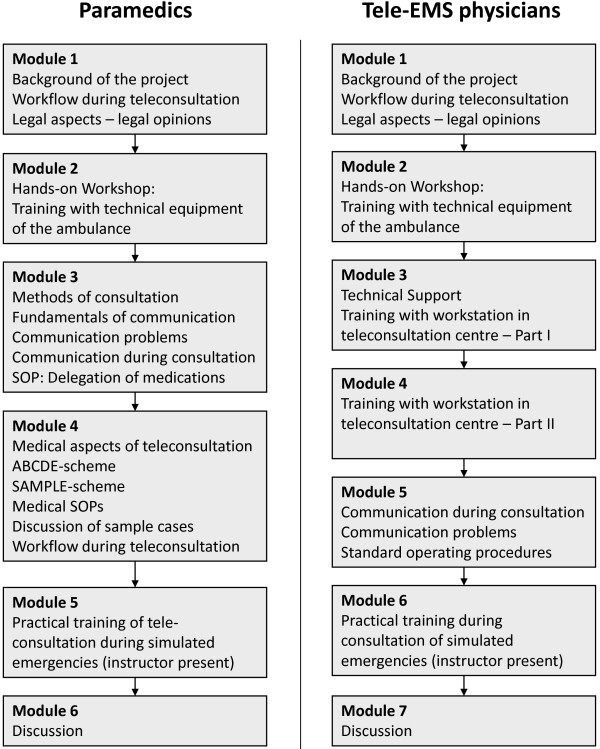
**Combined standardized training program for paramedics and tele**-**EMS physicians.** EMS, emergency medical service; SOP, standard operating procedure.

In all, 178 paramedics and 11 tele-EMS physicians completed a standardized eight-hour training program and performed training cases prior to the clinical implementation of this system (Figure 3). In Germany, paramedic training is a two-year program. All tele-EMS physicians had at least five years of clinical experience in anaesthesia and critical care as well as one year in the EMS. On August 1^st^ 2012, the system was implemented on three ambulances with two additional ambulances added later on (Table 2).

**Table 2:** Number of regular and telemedically assisted emergency missions

**District and observation period**	**Regular emergency missions**^**a**^	**Emergency missions with telepresence / teleconsultation**
Aachen 1 (city) 01.08.-31.08.2013	118	22 (18.6%)
Aachen 2 (rural) 01.08.-31.08.2013	75	3 (4.0%)
Heinsberg (rural) 01.08.-31.08.2013	76	6 (7.9%)
Düren (rural) 20.08.-31.08.2013	12	4 (33.3%)
Euskirchen (rural) 27.08.-31.08.2013	15	0^b^
Total number	296	35 (11.8%)

^a^ during operating time of teleconsultation centre: weekdays 7.30 am – 4.30 pm.

^b^ only technical testing performed.

### Data acquisition and analysis

The characteristics and case numbers of the four EMS districts were obtained from the EMS directors. Data on patient demographics, prehospital diagnoses, prehospital treatment, and the time points for the administration of medications and other measures were collected from the documentation protocols produced at the teleconsultation centre and from the paper-based EMS protocols. All medical protocols and daily reports of the teleconsultation centre were reviewed to search for unexpected events and observations, medical complications, technical failures and interpersonal communication problems. Prehospital time intervals were taken from the reports from the EMS dispatch centre. All patient-related data were retrieved by two faculty investigators, anonymized, and transferred to a spreadsheet (Excel 2003, Microsoft, Redmond, WA, USA). Descriptive statistical analyses were performed using SPSS Statistics 19 (SPSS, Chicago, IL, USA). Time intervals are presented as means plus standard deviations (SD). The frequencies for treatment measures and medications are described as medians and ranges.

## Results

Teleconsultation was provided by a pool of nine tele-EMS physicians during 35 (11.8%) of 296 emergency missions from August 1^st^ to 31^th^, 2012. Four of the consultation cases were secondary inter-hospital transfers. During these 35 missions, 31 patients were alert and oriented, and no patient refused teleconsultation; however, for 4 patients with a reduced level of consciousness, consent was assumed. The proportion of telemedically assisted missions differed considerably among the ambulances (Table 2). The mean duration of a consultation was 24.9 min (SD 12.5, range 3–58 min). Table 3 summarises the different indications and diagnoses. Teleconsultation was initiated in 29 (82.9%) missions with no physician present at the scene. Table 4 shows the different time points of initiation of teleconsultation. Diagnostic support (e.g., 12-lead ECG diagnoses, co-evaluation via video transmission) was provided by the tele-EMS physicians in 34 (97.1%) cases. Administration of medication was delegated to the paramedics in 21 (60%) consultations: 50 individual administrations of 16 different substances were ordered (range 1–7 per consultation, median 1), including 5 missions with orders for an opioid-based analgesia. The tele-physicians delegated other actions (e.g., intravenous access) that were performed by the paramedics in 22 missions (63%, range 1–4 per consultation, median 1), and in 13 (37%) missions, the choice of the admitting hospital was determined by the tele-EMS physician, who then also pre-notified the facility. No adverse medical events during or after teleconsultation were documented. In addition, no communication problems between the EMS team and the tele-EMS physician or other interpersonal negative effects were reported during the telephone based debriefings. Furthermore, no unexpected observations were made. From a technical point of view, all components functioned as expected despite some technical noises that were occasionally experienced due to interferences in the Bluetooth connection between the headsets and the transmission unit. Complete drop-outs of functionalities did not occur, but initiation of teleconsultation failed in one additional case (patient with moderate allergic reaction) due to the lack of an available mobile network inside an office building on the ground floor. None of the three mobile networks had a stable audio or data connection in this particular case.

**Table 3:** Prehospital diagnoses made during teleconsultations

**Diagnosis category**	**Number of missions**
Cardiac arrest^a^	2
Acute coronary syndrome	6
Circulatory emergency^b^	6
Respiratory emergency	2
Trauma	8
Neurological emergency	3
Gastrointestinal emergency	5
Other emergency	3

^a^ cardiopulmonary resuscitation performed.

^b^ no acute coronary syndrome/ST-elevation myocardial infarction included.

**Table 4:** Time points and circumstances of teleconsultation initiation

**Characteristics of teleconsultation**	**Number (%)**
Teleconsultation without an on-scene EMS physician	23 (65.7%)
Teleconsultation initiated while awaiting arrival of an EMS physician	3 (8.6%)
Teleconsultation initiated in the absence of an EMS physician; EMS physician requested after evaluation of the patient by the tele-EMS physician	3 (8.6%)
Teleconsultation initiated after arrival of an EMS physician	6 (17.1%)

*EMS* emergency medical service.

To describe some of the experiences and possible limitations in detail, we reviewed all of the teleconsultation protocols and chose three cases that illustrate different aspects of the teleconsultation system to present. In these cases, an EMS physician was not physically present at the emergency site when the teleconsultation was initiated.

### Patient 1: Diagnostic support and emergency treatment of acute coronary syndrome

Teleconsultation was initiated to request support for a 12-lead ECG diagnosis and evaluation of a 42-year-old male patient who complained of dizziness, nausea, and chest pain. The patient was already inside the parked ambulance. Using video transmission the tele-EMS physician assessed the patient as obese, pale, and diaphoretic but having stable vital signs. The 12-lead ECG was transmitted to the teleconsultation centre and did not indicate acute myocardial ischemia. However, risk factors for coronary artery disease were detected using the SAMPLE scheme (heavy smoker, stressful job, obesity, and no physical activity) and the tele-EMS physician assessed the patient as being at high risk for suffering from ACS. German EMS procedures dictate that ACS cases require an EMS physician; therefore, an EMS physician unit was summoned as advanced treatment was started. The tele-EMS physician ordered the following medications for administration by the paramedics: 0.8 mg nitroglycerin sublingual (provided only mild symptom relief), 500 mg acetylsalicylate acid and 5000 I.E. unfractionated heparin given intravenously. The regular EMS physician arrived 15 min after initiation of the teleconsultation and continued prehospital therapy. Meanwhile, the tele-EMS physician arranged admission to the chest pain unit at the local university hospital. The patient reached the hospital 33 min after the beginning of the teleconsultation.

### Patient 2: Analgesia for a patient with a displaced shoulder joint in a rural area

A 67-year-old female fell from the third rung of a ladder and dislocated her right shoulder joint. The ambulance crew initiated teleconsultation to request delegation of the administration of analgesics. After the initial evaluation using the ABCDE and SAMPLE scheme, the patient received 5 mg morphine intravenously. This reduced the pain level from a verbal patient-assessed score of 8 out of 10 to 3 out of 10. To prevent opioid-induced nausea, the paramedics also administered 62 mg of dimenhydrinate diluted in 500 ml balanced electrolyte solution. Although the EMS physician response unit was summoned in conjunction with the paramedic-staffed ambulance, it arrived 16 min after the beginning of the teleconsultation. Fentanyl was administered additionally by the EMS physician on site, and the teleconsultation was terminated.

### Patient 3: Witnessed sudden cardiac arrest

An 83-year-old male suddenly collapsed in a nursing home. Chest compressions and rescue breathing were administered by bystanders (nurse and urologist) until the ambulance arrived. The teleconsultation was initiated immediately after the paramedics arrived at the patient’s location. The tele-EMS physician confirmed the presence of an asystole and supervised the speed of chest compressions by watching the continuously transmitted rhythm ECG. A rate of 100–120 compressions/min was achieved throughout the resuscitation. Via the audio connection, the tele-EMS physician heard that the paramedics encountered initial problems in opening the airway for bag-valve mask ventilation and suggested primary supraglottic airway insertion (LMA Supreme, LMA Germany, Bonn, Germany), which enabled sufficient ventilation. An EMS physician reached the patient’s location 6 min after the teleconsultation began. Audio connection was continued, and the tele-EMS physician recommended early intraosseous access due to failed intravenous punctures. The return of spontaneous circulation (i.e., narrow complex tachycardia) was achieved 13 min after the arrival of the ambulance. During the transfer of the patient to the ambulance, the tele-EMS physician arranged the hospital admission, including pre-notification of the cardiology unit (i.e., the cath lab), the neuroradiology unit for a cerebral scan, and the intensive care unit.

## Discussion

After structured training of all prehospital medical staff, the implementation and clinical use of the telemedicine system was successfully realized. Teleconsultation between paramedics and remotely located physicians was feasible, and no medical adverse events were observed. With the described approach, immediate emergency therapy was possible well before the arrival of an EMS physician or arrival at the hospital. The system functioned technically as expected, although dependency on mobile phone networks is a predictable limitation.

The telemedical approach seemed to be efficient in cases when no physician was on-scene because the duration of the teleconsultation was considerably shorter than the duration of regular EMS physician missions such as those described by Schuster et al. in a metropolitan area [12]. Three of the five connected ambulances were located in rural EMS settings. In rural areas, the duration of an EMS physician mission may be even longer due to the long distances and the low number of EMS physician-staffed units available. Therefore, teleconsultation may be especially beneficial and efficient in such regions. The mean consultation time may become even shorter as paramedics and tele-EMS physicians become more accustomed to the system. For both the paramedics and the tele-EMS physicians, the described approach was a completely new responsibility. Therefore, the audio connection may have been continued in some cases despite the lack of a medical need. Interestingly, the proportion of telemedically assisted missions was not considerably lower in the urban setting (Aachen 1, Table 2) than in the rural settings. Overall, the rates of teleconsultation usage indicate a general acceptance of the system. However, the need for teleconsultation depended on the particular condition of the patient and in two ambulances, the observation period was so short that a conclusion regarding the utilization rate is not reliable. The main tasks accomplished by the tele-EMS physicians were diagnostic support and delegation of medication administration. This enabled the provision of earlier advanced therapy under real-time medical control, thereby likely improving patient safety in comparison to treatment by paramedics alone. In Germany, paramedics are only allowed to administer a few medications when an EMS physician is already on the way or alternatively they have to request an EMS physician. Whereas in other countries, measures such as intravenous access are considered basic procedures, in Germany, these actions are considered advanced measures. In cases where both the tele-EMS physician and later the EMS physician were involved, teleconsultation had the potential to shorten therapeutic intervals, as seen in our three case reports.

Although neither paramedics nor physicians had experience with teleconsultations, acceptance of this process was considered high because no communication problems or negative interpersonal effects were reported during the debriefings. Furthermore, no patient refused to consent to the teleconsultation, which indicates a high acceptance despite the fact that German citizens are not yet familiar with this approach.

For patient 1 the structured, software-supported assessment of the patient’s condition and medical history revealed relevant risk factors to the tele-EMS physician. This led to the summons of an on-scene EMS physician, which adheres to the national recommendations. While the EMS physician was en route, necessary intravenous administration of medications was started due to the availability and direction of the tele-EMS physician. However, if the transport had commenced directly after the successful intravenous access and all medications had been administered en route, the prehospital time could have been reduced. In the future, the process chain should be optimized to prevent prolongation of the prehospital time. Time savings can be achieved when organisational tasks are performed by the tele-EMS physician (e.g., as described in the cases 1 and 3). Pre-notification of the admitting facility was offered to the EMS teams in 37% of the cases. This allowed the paramedics and the EMS physician on-scene to focus on rapid treatment and transport. Additionally, in-hospital treatment processes can be prearranged. In cases of ST-segment elevation myocardial infarction and acute stroke, early pre-notification of the admitting hospital reduces in-hospital time intervals and ultimately improves patient outcomes [2,7]. However, in different organisational settings, personnel such as EMS dispatch centre staff could perform the pre-notification.

By German law, paramedics and nurses are not allowed to administer opioids without the direction of a physician. Therefore, prehospital analgesia is primarily administered by EMS physicians in regular EMS. In contrast, teleconsultation enabled earlier administration of opioids by paramedics without violating national law. Although analgesia was initiated much earlier than otherwise possible for patient 2, the administration of morphine alone did not lead to complete pain relief. Research has shown that single-agent analgesia with morphine is inferior to morphine combined with ketamine, but analgesia with morphine is safe and routinely used in paramedic-staffed EMS [13-16]. Since German paramedics do not administer opioids routinely, we intentionally delegated morphine as a single agent to be combined with antiemetics if necessary during this first phase of this project. However, medications were ordered in more than half of the cases in this study, including a total of 16 different substances. Interestingly, the tele-EMS physicians were conservative in ordering the administration of opioids. Dosages may increase as the use of the system continues.

Many of the cases using teleconsultation were not life threatening. But especially in life threatening cases teleconsultation may have the greatest potential for improving patient outcome. In every case that demonstrated the obvious need for an on-scene EMS physician, such as a patient with a reduced level of consciousness, one was sent by the dispatch centre. Teleconsultation was treated solely as an additional option. In cases that require complex, invasive procedures, such as pharmacologically assisted intubation, teleconsultation can only act as a support as shown in case 3. Qualified physician-staffed EMS offer better care and outcomes for patients in critical condition than some paramedic-staffed systems [17-19]. However, case 3 demonstrates that even an EMS physician can benefit from telemedical support. The EMS physician in case 3 was at the beginning of his prehospital practise. However, it is important to note that one cannot assume that without a telemedical recommendation a particular action would not have been performed eventually.

In the previous project entitled "Med-on-@ix," teleconsultation occurred between the on-scene EMS physician and the tele-EMS physician to test the technical and organisational concept [9-11]. In contrast, teleconsultations primarily occurred between the paramedics and the tele-EMS physician in the current project (Table 4). Clearly, not all aspects of an emergency can be addressed by teleconsultation, but in cases when a medical decision must be made, it may provide a beneficial alternative. Furthermore, it may offer an economic advantage because one tele-EMS physician can support multiple ambulances regardless of his/her physical location and that of the ambulance.

Projects with similar goals have been conducted for acute stroke and major trauma; however, their telemedical approaches were only researched using simulation, and the concepts were not transferred into clinical practise [20,21]. Furthermore, these projects were limited to specific emergency situations. In contrast, our described system enabled assistance in every kind of emergency. In the United States, two similar commercial systems are available. The LifeBot system (LifeBot, Phoenix, AZ, USA) enables vital data transmission, electronic documentation and decision support but does not have a video channel available and no parallelised data connection is integrated [22]. In contrast, the e-Bridge system (General Devices, Ridgefield, NJ, USA) allows a two-way videoconference from the ambulance additionally to the aforementioned applications and the system enables transmission with different technologies such as third generation cellular networks, Wi-Fi and radio networks [23,24]. No clinical studies regarding these systems have been published so their effectiveness remains unclear.

The TemRas system demonstrated a noticeably improved technical performance compared with that of the precursor system, in which drop-out rates of up to 20% were detected [9]. Furthermore, the video transmission performance was considerably better than that of a project that specifically equipped ambulances for acute stroke care [25]. However, teleconsultation in the current study failed in one case. Dependency on mobile networks seems to be the major but predictable limitation of mobile telemedicine systems. Even with the integrated fall-back solution of using different networks, stable transmission cannot be guaranteed. One possible solution for such problems may be the integration of satellite-based connection. This was not integrated due to the high associated costs. However, in the described case of non-availability of any mobile network satellite-based communication would not have been possible. Nevertheless, this technology should be evaluated in the future.

### Limitations

This study presents the initial results of the implementation phase of a newly developed telemedicine system. The selection of the EMS districts was not randomized, but was based on local cooperation, which represents a potential bias. Issues such as the rate of teleconsultation, rate of complications and outcome effects should be evaluated in larger future studies. However, our data demonstrated that teleconsultation can assist in a broad spectrum of emergencies. The usage of the system may depend not only on medical necessity but possibly also on the medical team's attitude towards the system. We cannot exclude the possibility that only the best medically or technologically trained paramedics used the system during this initial phase. Indeed, guidelines for the use of telemedical support have not yet been established. Nevertheless, the fact that no medical complications were reported as a result of teleconsultation indicates that it is likely safe and can enable quicker treatment for patients.

## Conclusions

The concept of telemedically assisted paramedic care was successfully implemented in five ambulances from four different EMS. This provides medical assistance to bridge the time between the arrival of the paramedics and the arrival of an EMS physician, and it can enable the provision of advanced treatment beyond that which is allowed with existing protocols without violating the laws. The approach seems to be well accepted, safe, and efficient; however a larger study is required to determine the rate of complications and outcome data. A comparison with the services provided by regular EMS should be conducted in both urban and rural EMS districts.

## Competing interests

The study was conducted within the joint research project “TemRas“ funded by the European Union and the Ministry of Innovation, Science and Research of Northrhine Westphalia, Germany (MIWF), Project-No.: PtJ-Az. 0909im002b, Funding code 005-1003-0034. Philips Healthcare (Hamburg, Germany) and P3 communications (Aachen, Germany) contributed their own financial resources. The funders had no role in the study design, data collection and analysis, decision to publish, or preparation of the manuscript. No author had financial relationships with or other non-financial dependencies on the funding sponsors.

## Authors' contributions

SB, JCB and RR conceived the initial idea of the study. All authors were engaged in the development and testing of the system. FH, DW, SB, BV and MC performed data collection. MC, BV and SB performed the descriptive statistical analysis. SB, JCB, MC, MTS, FH and RR performed data interpretation. SB, BV, FH, JCB, SKB and RR drafted the manuscript and DW, MTS, SKB and MC performed literature searches. MC, DW, MTS revised the manuscript critically. All authors read and approved the final manuscript.
